# Synthesis and Preclinical Evaluation of Three Novel ^68^Ga-Labeled Bispecific PSMA/FAP-Targeting Tracers for Prostate Cancer Imaging

**DOI:** 10.3390/molecules28031088

**Published:** 2023-01-21

**Authors:** Arsyangela Verena, Zhengxing Zhang, Hsiou-Ting Kuo, Helen Merkens, Jutta Zeisler, Ryan Wilson, Shreya Bendre, Antonio A. W. L. Wong, François Bénard, Kuo-Shyan Lin

**Affiliations:** 1 Department of Molecular Oncology, BC Cancer Research Institute, Vancouver, BC V5Z1L3, Canada; 2 Department of Radiology, University of British Columbia, Vancouver, BC V5Z1M9, Canada; 3 Department of Functional Imaging, BC Cancer, Vancouver, BC V5Z4E6, Canada

**Keywords:** prostate-specific membrane antigen, fibroblast activation protein, bispecific radiotracers, PET/CT, Gallium-68

## Abstract

Tumor heterogeneity limits the efficacy and reliability of monospecific radiopharmaceuticals in prostate cancer diagnosis and therapy. To overcome this limitation and improve lesion detection sensitivity, we developed and evaluated three bispecific radiotracers that can target both prostate-specific membrane antigen (PSMA) and fibroblast activation protein (FAP), which are the two key proteins overexpressed in prostate cancer. Three FAP-targeting ligands with various linker lengths were synthesized through multistep organic synthesis, and then connected to the PSMA-targeting motif. IC_50_(PSMA) and IC_50_(FAP) values of Ga-complexed bispecific ligands, Ga-AV01017, Ga-AV01030, and Ga-AV01038 were 25.2–71.6 and 1.25–2.74 nM, respectively. The uptake values in PSMA-expressing LNCaP tumor xenografts were 4.38 ± 0.55, 5.17 ± 0.51, and 4.25 ± 0.86 %ID/g for [^68^Ga]Ga-AV01017, [^68^Ga]Ga-AV01030, and [^68^Ga]Ga-AV01038, respectively, which were lower than the monospecific PSMA-targeting tracer [^68^Ga]Ga-HTK03041 (23.1 ± 6.11 %ID/g). The uptake values in FAP-expressing HEK293T:hFAP tumor xenografts were 2.99 ± 0.37, 3.69 ± 0.81, 3.64 ± 0.83 %ID/g for [^68^Ga]Ga-AV01017, [^68^Ga]Ga-AV01030, and [^68^Ga]Ga-AV01038, respectively, which were also lower than the monospecific FAP-targeting tracer, [^68^Ga]Ga-FAPI-04 (12.5 ± 2.00 %ID/g). We observed that the bispecific tracers had prolonged blood retention, in which tracers with a longer linker tend to have a higher blood uptake and lower tumor uptake. Further investigations are needed to optimize the linker selection to generate promising bispecific PSMA/FAP-targeting tracers for prostate cancer imaging.

## 1. Introduction

Prostate-specific membrane antigen (PSMA), a type II transmembrane glycoprotein also known as glutamate carboxypeptidase II, has enzymatic functions to cleave terminal glutamate [1,2]. PSMA has been shown to be overexpressed in prostate tumor and the neovasculature of other type of cancer, such as colon and renal cancers [3], making it an attractive imaging and therapeutic target. Various PSMA-targeting radioligands have been developed and three of them have been approved by the US FDA: [^18^F]DCFPyL and [^68^Ga]Ga-PSMA-11 for prostate cancer imaging [4,5] and [^177^Lu]Lu-PSMA-617 for prostate cancer radioligand therapy [6].

Although [^177^Lu]Lu-PSMA-617 has been proven to be effective in treating metastatic castration-resistant prostate cancer (mCRPC) patients [7], some patients have lesions with heterogeneous expression, or even no expression of PSMA [8,9], limiting their eligibility in benefiting from PSMA-directed therapy. Moreover, studies have shown that lesions that are PSMA-negative, such as neuroendocrine prostate cancer (NEPC), are prone to being more aggressive and metabolically active, shown by high [^18^F]FDG uptake [10]. Due to the intrapatient heterogeneous PSMA expression in prostate cancer, several PSMA-negative lesions could be missed by PSMA-targeting radioligands which could lead to worse overall survival [11,12]. Therefore, new strategies should be considered to improve lesion detection for this patient cohort.

As a member of type II transmembrane serine proteases, fibroblast activation protein (FAP) acts on various hormones and extracellular matrix components [13,14]. FAP is found to be highly expressed in activated fibroblasts, such as during wound healing [15], and is essentially absent or lowly expressed in normal adult cells [16]. Many studies have demonstrated that FAP is expressed by the cancer-associated fibroblasts (CAFs) that occupy the tumor microenvironment (TME) [17,18], and is overexpressed in 90% of epithelial tumors, including colon and breast cancers [19,20]. FAP expression is associated with worse prognosis as FAP supports tumor migration [21], angiogenesis [22], metastasis via matrix remodeling in TME [23], and immunomodulatory function [24]. A study by Kesch et al. [25] showed that there is a significant rise in FAP expression throughout the progression of prostate cancer, in which men with advanced CRPC have the highest number of FAP-positive lesions. Seeing the potential of FAP as a diagnostic and therapeutic target, there have been many studies evaluating new FAP targeting for imaging and radioligand therapy. For instance, [^68^Ga]Ga-FAPI-04, a quinoline-based FAP-targeting tracer [26], has been shown to have faster pharmacokinetics, higher tumor uptake, and superior tumor-to-background contrast in patients with various cancers when compared with [^18^F]FDG [27]. Although several novel FAP-targeting radiotherapeutic agents have been developed, such as [^90^Y]Y-FAPI-04 [26], [^177^Lu]Lu-DOTA.SA.FAPi [28], and [^177^Lu]Lu-FAPI-46 [29], their tumor-retention time and efficacy in clinical investigation have been disappointing.

**Figure 1:**
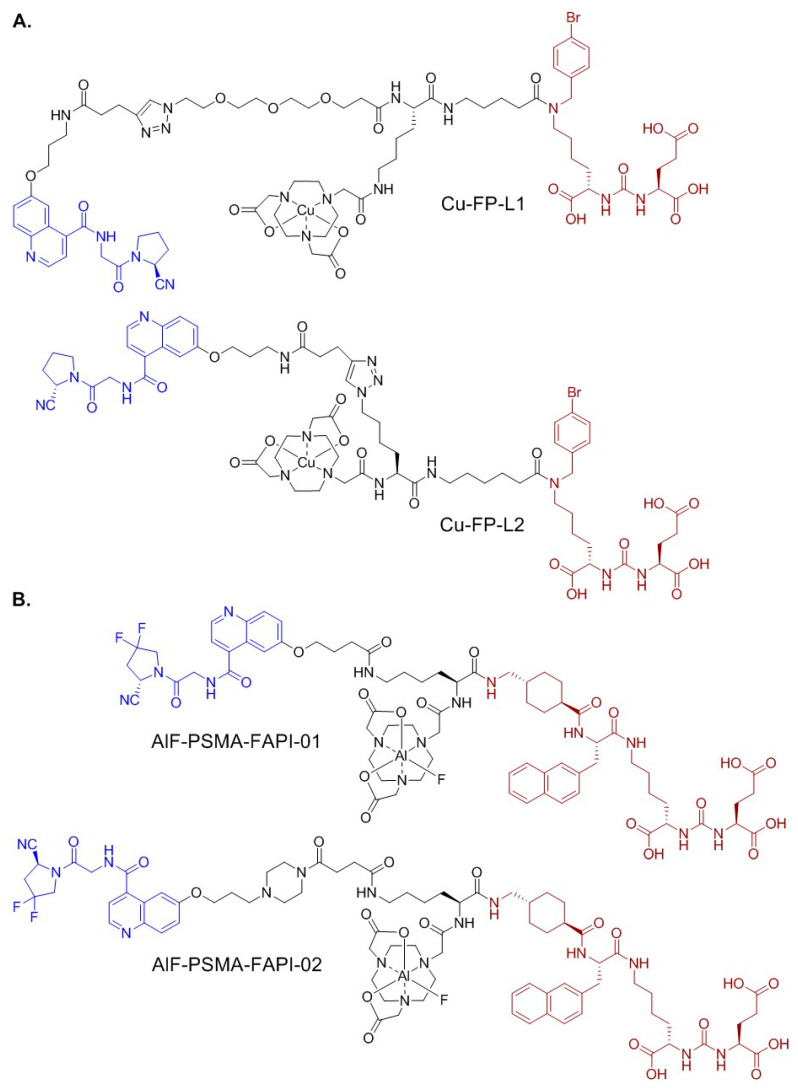
Chemical structures of (**A**) Cu-FP-L1 and Cu-FP-L2; and (**B**) AlF-PSMA-FAPI-01 and AlF-PSMA-FAPI-02. The PSMA- and FAP-targeting pharmacophores are shown in brown and blue, respectively.

Previous studies have shown the potential of using bispecific radioligands targeting PSMA and other overexpressed proteins, such as gastrin-releasing peptide receptor (GRPR), to improve tumor targeting and increase the detection sensitivity of prostate cancer imaging [30,31]. Here, our goal is to develop PSMA/FAP bispecific radioligands with comparable or even higher tumor uptake compared to the PSMA- and FAP-targeting monospecific tracers. Previously, Boinapally et al. [32] reported two ^64^Cu-labelled PSMA/FAP bispecific tracers, [^64^Cu]Cu-FP-L1 and [^64^Cu]Cu-FP-L2 (Figure 1A), which showed high and specific uptake in both FAP- and PSMA-expressing tumor models. However, no head-to-head comparison of their bispecific tracers with the FAP- or PSMA-targeting monospecific tracer was reported. Hu et al. [33] reported the development of two ^18^F-labeled PSMA/FAP bispecific tracers, [^18^F]AlF-PSMA-FAPI-01 and [^18^F]AlF-PSMA-FAPI-02 (Figure 1B), and both showed higher uptake in PSMA- and FAP-expressing tumor models when compared with the PSMA- and FAP-targeting monospecific tracers, respectively. Although promising results were obtained from these two reports, their use of NOTA as the radioisotope chelator excludes the labeling of these reported PSMA/FAP bispecific ligands with the commonly used radiotherapeutic metals such as ^177^Lu and ^90^Y.

To expand the potential usage of bispecific PSMA/FAP-targeting ligands for radiotherapeutic applications, we chose DOTA as our chelator, which has theranostic capabilities to label both diagnostic isotopes, such as ^68^Ga, and therapeutic isotopes, such as ^177^Lu. This will enable us to use the same ligands for diagnostic applications and ensure the pharmacokinetics of diagnostic and radiotherapeutic agents are comparable as they are derived from the same PSMA/FAP-targeting ligands.

**Figure 2:**
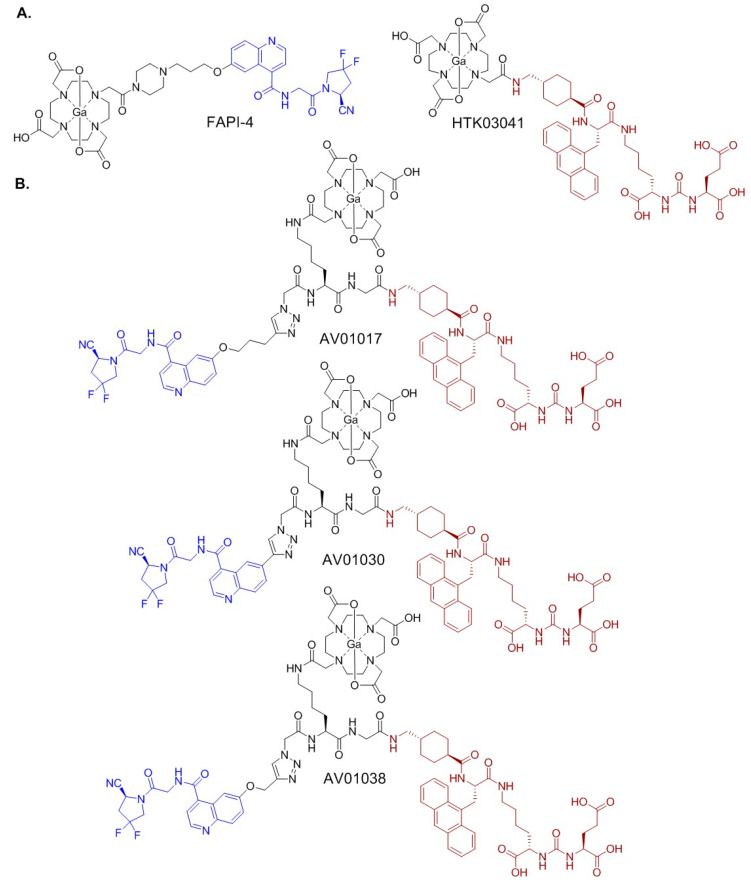
Chemical structures of (**A**) PSMA-targeting [^68^Ga]Ga-HTK03041 and FAP-targeting [^68^Ga]Ga-HTK03041; and (**B**) bispecific PSMA/FAP-targeting ^68^Ga-labeled AV01017, AV01030, and AV01038. The PSMA- and FAP-targeting pharmacophores are shown in brown and blue, respectively.

Here we report the design, synthesis, and evaluation of three ^68^Ga-labeled DOTA-conjugated bispecific PSMA/FAP tracers (Figure 2). The PSMA binding motif of AV01017, AV01030, and AV01038 was based on our previously reported [^68^Ga]Ga-HTK03041 [34], and their FAP-targeting motif was derived from [^68^Ga]Ga-FAPI-04. The difference between these three tracers is the length of their linker between the quinoline and the triazole with ring null, -O-CH_2_-, and -O-(CH_2_)_3_- for ^68^Ga-labeled AV01030, AV01038, and AV01017, respectively. Their potential of the tracers for prostate cancer imaging was evaluated by an in vitro competition binding assay, PET imaging, and ex vivo biodistribution studies in preclinical PSMA-expressing LNCaP and FAP-expressing HEK293T:hFAP tumor models in mice. The results were then compared with those of the corresponding monospecific tracers, [^68^Ga]Ga-HTK03041 and [^68^Ga]Ga-FAPI-04.

## 2. Results

### 2.1. Synthesis of PSMA/FAP Bispecific Ligands

AV01017, AV01030, and AV01038 were synthesized on solid phase. Detailed synthetic procedures and characterizations are provided in the Supplementary Materials (Supplementary Materials, Table S1). Briefly, Lys(azidoacetic acid-Lys(ivDde)-Gly-tranexamic acid-Ala(9-Anth))-urea-Glu(OtBu)-OtBu was first constructed on solid phase, followed by the click addition of the alkyne-containing FAP-targeting motif: compound **5** for AV01017, Compound **9** for AV01030, and compound **13** for AV01038. The amino group at the Lys side chain was then deprotected and coupled with the DOTA chelator. The DOTA-conjugated ligands were then cleaved off from resin and purified by HPLC.

**Scheme 1:**
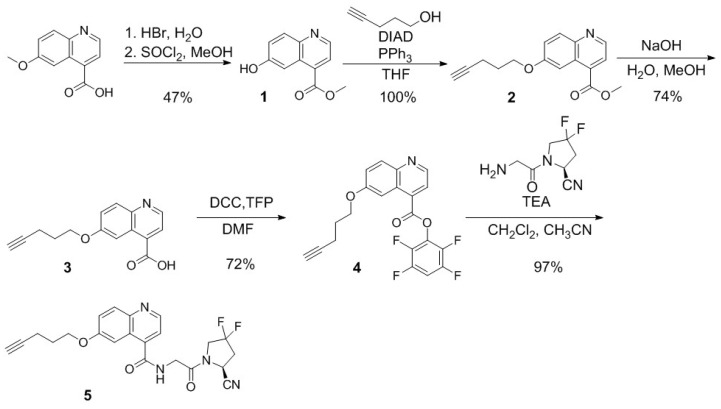
Synthesis of (*S*)-*N*-(2-(2-cyano-4,4-difluoropyrrolidin-1-yl)-2-oxoethyl)-6-(pent-4-yn-1-yloxy)quinoline-4-carboxamide (**5**).

**Scheme 2:**
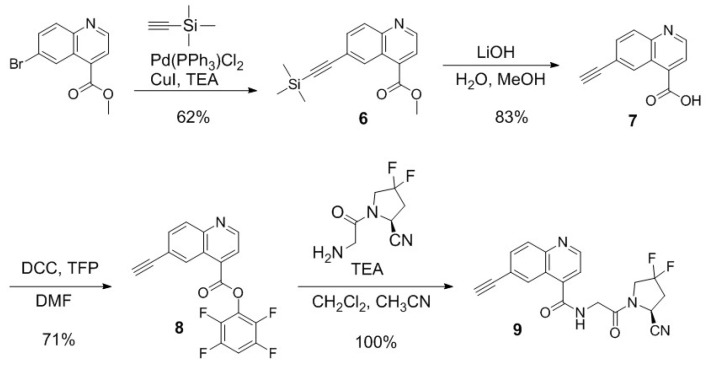
Synthesis of (*S*)-*N*-(2-(2-cyano-4,4-difluoropyrrolidin-1-yl)-2-oxoethyl)-6-ethynylquinoline-4-carboxamide (**9**).

**Scheme 3:**
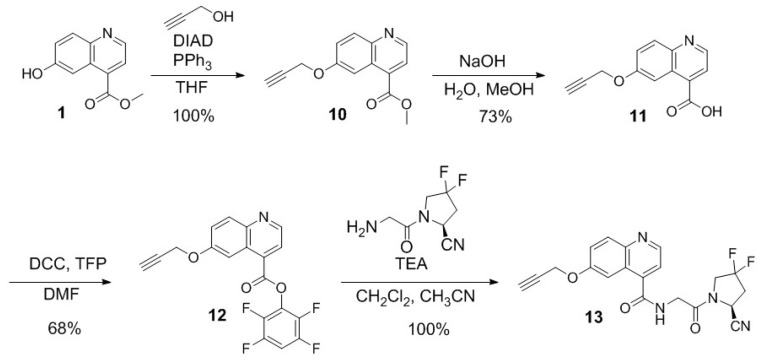
Synthesis of (*S*)-*N*-(2-(2-cyano-4,4-difluoropyrrolidin-1-yl)-2-oxoethyl)-6-(prop-2-yn-1-yloxy)quinoline-4-carboxamide (**13**).

The synthesis of compounds **5**, **9**, and **13** are depicted in Scheme 1, Scheme 2 and Scheme 3, respectively, and detailed synthetic procedures and characterizations for the final products and intermediates are provided in the Supplementary Materials. For the preparation of compound **5** (Scheme 1), quininic acid was demethylated in 48% HBr solution, followed by esterification in methanol in the presence of thionyl chloride to obtain **1** in 47% yield. Mitsunobu coupling between compound **1** and 4-pentyn-1-ol resulted in **2** in 100% yield. Compound **3** was obtained in 74% yield by the hydrolysis of **2** with NaOH in a mixture of water and methanol. Esterification of compound **3** with tetrafluorphenol (TFP) led to compound **4** in 72% yield. Compound **5** was obtained in 97% yield by coupling the activated ester **4** with (*S*)-1-(2-aminoacetyl)-4,4-difluoropyrrolidine-2-carbonitrile.

The synthesis of compound **9** is depicted in Scheme 2. The Sonogashira reaction was performed for coupling methyl 6-bromoquinoline-4-carboxylate and ethynyltrimethylsilane to obtain compound **6** in 62% yield. Silane deprotection and ester hydrolysis were performed simultaneously with LiOH in a mixture of water and methanol to afford compound **7** in 83% yield. Compound **7** was subsequently activated with tetrafluorophenol to afford the activated ester **8** in 71% yield. The desired compound **9** was obtained in 100% yield by coupling the activated ester **8** with (*S*)-1-(2-aminoacetyl)-4,4-difluoropyrrolidine-2-carbonitrile.

The synthetic procedures for the preparation of compound **13** are provided in Scheme 3. Mitsunobu coupling of compound **1** and 2-propyn-1-ol resulted in **10** in 100% yield. Compound **10** was hydrolyzed with NaOH in a mixture of water and methanol to obtain compound **11** in 73% yield. Activation of compound **11** with tetrafluorophenol resulted in compound **12** in 68% yield. Compound **13** was obtained in 100% yield by coupling the activated ester **12** with (*S*)-1-(2-aminoacetyl)-4,4-difluoropyrrolidine-2-carbonitrile.

Detailed syntheses and characterizations of nonradioactive Ga- and ^68^Ga-complexed AV01017, AV01030, and AV01038 are provided in the Supplementary Materials (Supplementary Materials, Tables S2 and S3). Nonradioactive Ga-complexed AV01017, AV01030, and AV01038 were obtained in 25–89% yields, and their ^68^Ga-labeled analogs were obtained in 43–60% decay-corrected radiochemical yields with >74 GBq/µmol molar activity and >99% radiochemical purity.

### 2.2. Binding Affinity

**Figure 3:**
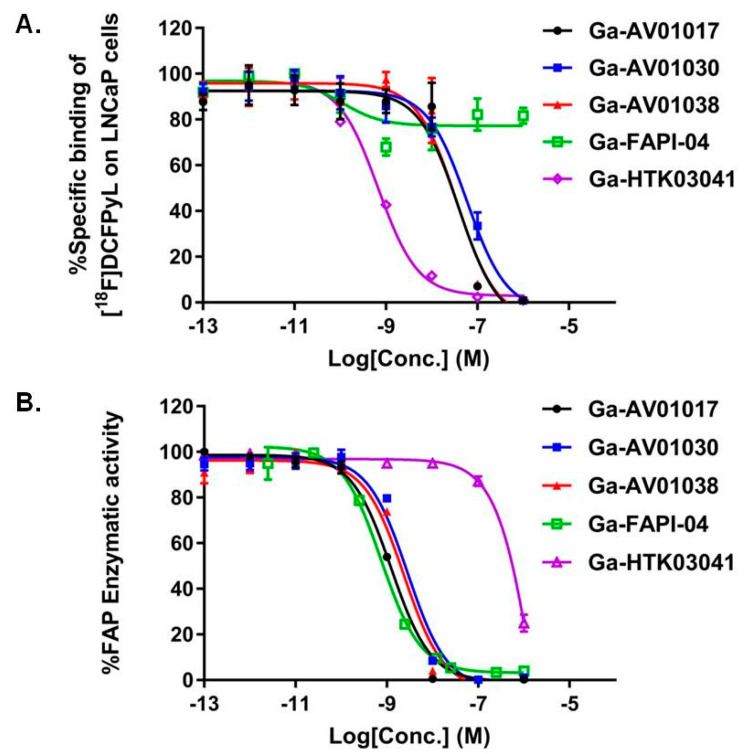
(**A**) Displacement curves of [^18^F]DCFPyL by Ga-AV01017, Ga-AV01030, Ga-AV01038, Ga-FAPI-04, and Ga-HTK03041 generated using PSMA-expressing LNCaP cells; (**B**) Fluorescence curve of FAP enzymatic activity on Suc-Gly-Pro-AMC substrate with inhibition by Ga-AV01017, Ga-AV01030, Ga-AV01038, Ga-FAPI-04, and Ga-HTK03041.

The binding affinities of Ga-AV01017, Ga-AV01030, Ga-AV01038, Ga-HTK03041, and Ga-FAPI-04 to PSMA were measured by a cell-based binding assay using PSMA-expressing LNCaP prostate cancer cells. The nonradioactive Ga-complexed standards, except Ga-FAPI-04, inhibited the binding of [^18^F]DCFPyL to LNCaP cells in a dose-dependent manner (Figure 3A). The calculated IC_50_(PSMA) values for Ga-AV01017, Ga-AV01030, Ga-AV01038, Ga-HTK03041, and Ga-FAPI-04 were 25.2 ± 10.7, 71.6 ± 23.0, 29.4 ± 25.2, 0.76 ± 0.12 and >1000 nM, respectively (*n* = 3).

The binding affinities of Ga-AV01017, Ga-AV01030, Ga-AV01038, Ga-HTK03041, and Ga-FAPI-04 to human FAP were measured by an enzyme-inhibition assay using Suc-Gly-Pro-AMC as the FAP substrate. The human FAP enzymatic activity on the substrate was inhibited by Ga-complexed standards in a dose-dependent manner (Figure 3B). The calculated IC_50_(FAP) values for Ga-AV01017, Ga-AV01030, Ga-AV01038, Ga-HTK03041, and Ga-FAPI-04 were 1.25 ± 0.39, 2.74 ± 0.33, 2.31 ± 0.13, 2010 ± 585, and 1.03 ± 0.4 nM, respectively (*n* = 3).

### 2.3. PET Imaging and Biodistribution Studies

**Figure 4:**
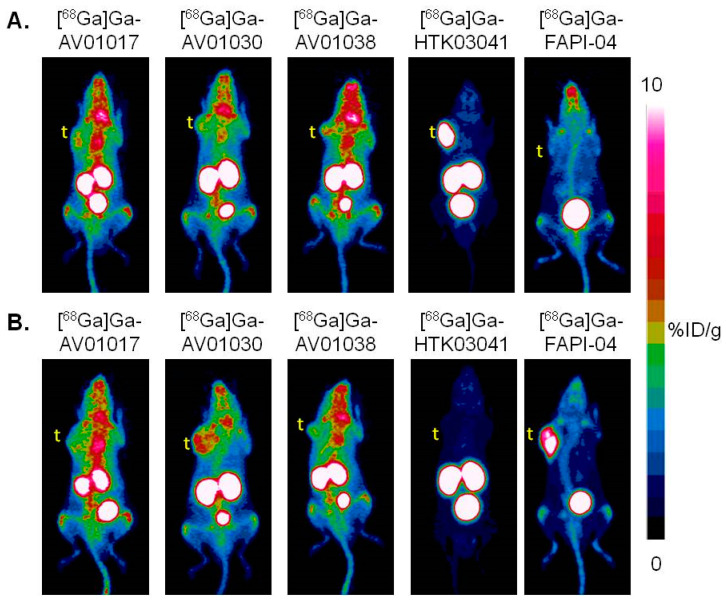
Representative maximum intensity projection PET images of [^68^Ga]Ga-AV01017, [^68^Ga]Ga-AV01030, [^68^Ga]Ga-AV01038, [^68^Ga]Ga-HTK03041, and [^68^Ga]Ga-FAPI-04 acquired at 1 h post-injection in mice bearing (**A**) LNCaP tumor xenografts and (**B**) HEK293T:hFAP tumor xenografts. t: tumor.

Representative PET images acquired at 1 h post injection using [^68^Ga]Ga-AV01017, [^68^Ga]Ga-AV01030, [^68^Ga]Ga-AV01038, [^68^Ga]Ga-HTK03041, and [^68^Ga]Ga-FAPI-04 are provided in Figure 4. All the radiotracers were excreted primarily through the renal pathway. All the bispecific tracers ([^68^Ga]Ga-AV01017, [^68^Ga]Ga-AV01030, and [^68^Ga]Ga-AV01038) had significantly higher background and heart uptake compared to the monospecific tracers ([^68^Ga]Ga-HTK03041 and [^68^Ga]Ga-FAPI-04). LNCaP tumor xenografts were clearly visualized by [^68^Ga]Ga-HTK03041 with an excellent contrast, barely visualized by the bispecific tracers ([^68^Ga]Ga-AV01017, [^68^Ga]Ga-AV01030, [^68^Ga]Ga-AV01038), and not visualized by [^68^Ga]Ga-FAPI-04 (Figure 4A). For HEK293T:hFAP tumor xenografts, they were clearly visualized by [^68^Ga]Ga-FAPI-4 with an excellent contrast and by [^68^Ga]Ga-AV01030 with a good contrast, barely visualized by [^68^Ga]Ga-AV01017 and [^68^Ga]Ga-AV01038, and not visualized by [^68^Ga]Ga-HTK03041 (Figure 4B). All tracers, except [^68^Ga]Ga-HTK03041 have bone and joint uptake, which is commonly observed for FAP-targeting tracers. High thyroid uptake was also observed in [^68^Ga]Ga-AV01017, [^68^Ga]Ga-AV01030, and [^68^Ga]Ga-AV01038 PET images. There was a high kidney uptake in mice injected with the bispecific tracers and [^68^Ga]Ga-HTK03041, but not in mice injected with [^68^Ga]Ga-FAPI-04.

**Figure 5:**
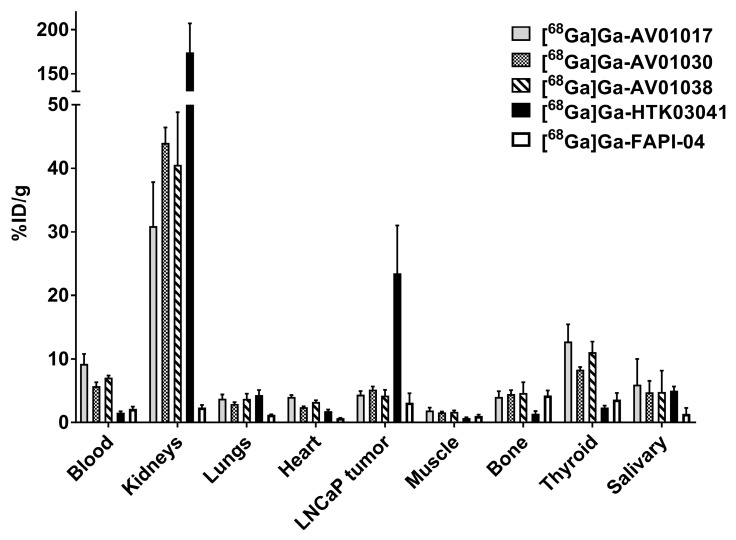
Biodistribution of [^68^Ga]Ga-AV01017, [^68^Ga]Ga-AV01030, [^68^Ga]Ga-AV01038, [^68^Ga]Ga-HTK03041, and [^68^Ga]Ga-FAPI-04 in LNCaP tumor-bearing mice.

Biodistribution studies were conducted at 1 h post injection with ^68^Ga-labeled AV01017, AV01030, AV01038, HTK03041, and FAPI-04 in LNCaP tumor-bearing mice (Figure 5 and Table S4, Supplementary Materials). The results were consistent with the observation from their PET images. Tumor uptake values for [^68^Ga]Ga-AV01017, [^68^Ga]Ga-AV01030, [^68^Ga]Ga-AV01038, [^68^Ga]Ga-HTK03041, and [^68^Ga]Ga-FAPI-04 were 4.38 ± 0.55, 5.17 ± 0.51, 4.25 ± 0.86, 23.1 ± 6.11, and 3.15 ± 1.43 %ID/g, respectively. All the bispecific tracers have statistically significantly higher heart and blood uptake than the monospecific [^68^Ga]Ga-HTK03041 and [^68^Ga]Ga-FAPI-04 (blood uptake: 5.75–9.24 vs. 1.43–2.16 %ID/g, *p* < 0.05; heart uptake: 2.41–4.04 vs. 0.70–1.82 %ID/g, *p* < 0.05). Although not statistically significant, the bispecific tracers with a longer linker tend to have a higher blood uptake value (9.24 ± 1.55 %ID/g for [^68^Ga]Ga-AV01017; 7.07 ± 0.31%ID/g for [^68^Ga]Ga-AV01038; 5.75 ± 0.59 %ID/g for [^68^Ga]Ga-AV01030) and a lower tumor-to-blood ratio (0.48 ± 0.11 for [^68^Ga]Ga-AV01017; 0.60 ± 0.13 for [^68^Ga]Ga-AV01038; 0.89 ± 0.03 for [^68^Ga]Ga-AV01030). The bispecific tracers also had significantly higher uptake values in muscle and thyroid when compared to those of monospecific radiotracers (*p* < 0.05).

**Figure 6:**
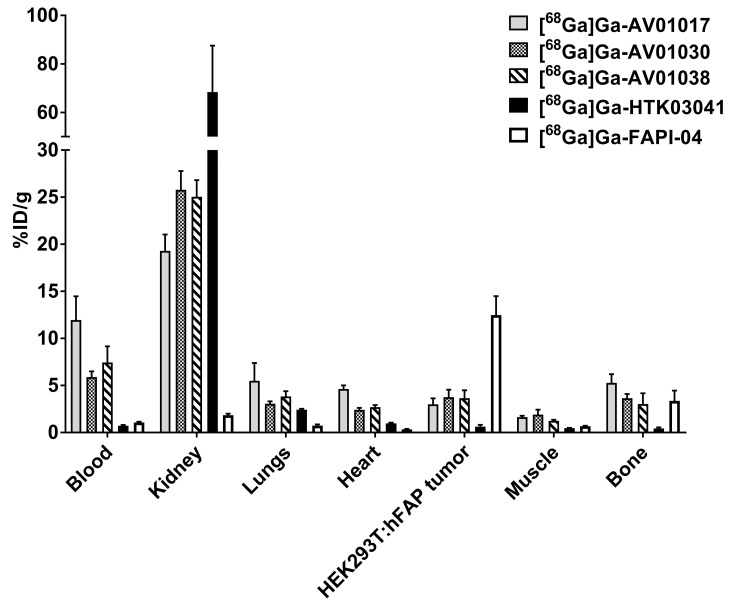
Biodistribution of [^68^Ga]Ga-AV01017, [^68^Ga]Ga-AV01030, [^68^Ga]Ga-AV01038, [^68^Ga]Ga-HTK03041, and [^68^Ga]Ga-FAPI-04 in HEK293T:hFAP tumor-bearing mice.

Biodistribution studies were also conducted at 1 h post injection with [^68^Ga]Ga-AV01017, [^68^Ga]Ga-AV01030, [^68^Ga]Ga-AV01038, [^68^Ga]Ga-HTK03041, and [^68^Ga]Ga-FAPI-04 in HEK293T:hFAP tumor-bearing mice (Figure 6 and Table S5, Supplementary Materials). Tumor uptake values for [^68^Ga]Ga-AV01017, [^68^Ga]Ga-AV01030, [^68^Ga]Ga-AV01038, [^68^Ga]Ga-HTK03041, and [^68^Ga]Ga-FAPI-04 were 2.99 ± 0.37, 3.69 ± 0.81, 3.64 ± 0.83, 0.62 ± 0.19, and 12.5 ± 2.00 %ID/g, respectively. There was a very low tumor uptake in mice injected with [^68^Ga]Ga-HTK03041, showing very minimal FAP expression in this tumor model. The uptake levels of these tracers on the major organs and tissues are consistent with the trends observed in the LNCaP tumor-baring mice (Figure 5 and Supplemental Table S4).

## 3. Discussion

Both PSMA and FAP are promising biomarkers of prostate cancer and many radioligands have been developed to target these two membrane proteins for imaging and therapy. However, the detection sensitivities of these radiotracers are strongly dependent on the expression levels and heterogeneities of these two biomarkers in different disease stages and between individuals [8,9]. Despite many effective PSMA-targeting radiotherapeutic agents being developed, patients with low to no PSMA expression are not eligible for these emerging PSMA-targeted radioligand therapies and still have very limited treatment options. Since FAP and PSMA are both expressed in prostate cancer and other cancers such as pancreatic cancer [25,35,36,37,38], the use of PSMA/FAP bispecific radioligands is expected to increase lesion detection sensitivity and treatment efficacy. The ultimate goal of this reported research is to develop ^68^Ga-labeled PSMA/FAP bispecific tracers with comparable or higher tumor uptake when compared to the monospecific tracers, [^68^Ga]Ga-FAPI-04 and [^68^Ga]Ga-HTK03041, a PSMA-targeting tracer previously reported by our group [34].

To date there have been only two reports on the development of PSMA/FAP bispecific tracers for imaging (Figure 1) [32,33]. Both reports used the NOTA chelator for radioisotope complexation: one for ^64^Cu [32] and the other for Al^18^F [33]. To the best of our knowledge, there has been no report on the development of ^68^Ga-labeled PSMA/FAP bispecific tracers despite the growing popularity and increased accessibility of clinical ^68^Ga generators. Although NOTA can be used for labeling ^68^Ga as well, it cannot be used for labeling the common and effective radiotherapeutic nuclides such as ^90^Y and ^177^Lu. Therefore, for the current research, we chose DOTA as the chelator for ^68^Ga labeling as DOTA is a widely used chelator and is effective for labeling with many diagnostic (such as ^68^Ga, ^152^Tb, and ^111^In) and radiotherapeutic radionuclides (such as ^149^Tb, ^90^Y, and ^177^Lu).

We selected the pharmacophores of [^68^Ga]Ga-FAPI-04 (in blue, Figure 2) and [^68^Ga]Ga-HTK03041 (in brown, Figure 2) for the design of our PSMA/FAP bispecific tracers as they have high affinity for FAP and PSMA, respectively [26,34]. These two pharmacophores are separated by an azidoacetic-Lys-Gly linker (Figure 2) to minimize the interaction of both pharmacophores as such interaction might interfere with the bindings of both pharmacophores to their respective targets. The PSMA-targeting pharmacophore (Lys(tranexamic acid-Ala(9-Anth))-urea-Glu and the linker (azidoacetic acid-Lys-Gly) was constructed directly on solid phase using the commercially available amino acids. While the DOTA chelator was coupled to the amino group on the Lys side chain, the alkyne-containing FAP-targeting motif was coupled to the azido group via the formation of a triazole ring by click chemistry. For AV01030, the triazole ring is directly linked to the quinoline ring of the FAP-targeting pharmacophore. For AV01038 and AV01017, there are additional -O-CH_2_- and -O-(CH_2_)_3_-, respectively, to separate the triazole ring and the quinoline ring of the FAP-targeting pharmacophore. This allowed us to investigate the effect of the additional linker and its length on the binding affinity and pharmacokinetics of the resulting bispecific tracers.

The enzymatic assay (Figure 3B) confirmed that the FAP binding affinities of our bispecific ligands (IC_50_ = 1.25–2.74 nM) were comparable to that of Ga-FAPI-04 (IC_50_ = 1.03 nM). To investigate if the PSMA-targeting pharmacophore has any effect on the overall FAP binding of our bispecific ligands, we also measured the FAP binding affinity of Ga-HTK03041. The very weak binding affinity of Ga-HTK03041 (IC_50_ = 2010 ± 585 nM) suggests that the potent FAP binding affinity of our bispecific ligands is contributed mainly by the FAPI-04 pharmacophore.

Unlike the comparable FAP binding affinity of bispecific ligands and the monospecific Ga-FAPI-4, the PSMA binding affinities of bispecific ligands (IC_50_ = 25.2–71.6 nM) were inferior to that of Ga-HTK03041 (IC_50_ = 0.76 nM). Although Ga-FAPI-04 has minimal binding affinity to PSMA (IC_50_ > 1000 nM), the addition of its pharmacophore and a linker clearly interferes the overall binding of the bispecific ligands to PSMA. Such interference seems to decrease with the increased linker length between the triazole ring and the FAP-targeting pharmacophore as the IC_50_(PSMA) values for Ga-AV01030, Ga-AV01038, and Ga-AV01017 are 71.6, 29.4, and 25.2 nM, respectively.

PET imaging and biodistribution studies (Figure 4, Figure 5 and Figure 6 and Supplemental Tables S4 and S5) revealed that the bispecific tracers retained the characteristics of the monospecific tracers as high uptake was observed in common off-targets of PSMA-targeting (kidneys and salivary glands) and FAP-targeting tracers (joints and salivary glands). However, the bispecific tracers showed much lower uptake values in LNCaP and HEK293T:hFAP tumor xenografts when compared to those of the monospecific tracers, [^68^Ga]Ga-HTK03041 and [^68^Ga]Ga-FAPI-04, respectively. Unlike the fast blood clearance of the monospecific tracers, the bispecific tracers had a much longer retention in blood. It seems that the blood retention was positively correlated to the length of the linker between the triazole ring and the FAP-targeting pharmacophore as the blood uptake values for Ga-AV01030, Ga-AV01038, and Ga-AV01017 are 5.68–5.75, 7.07–7.45, and 9.24–11.9 %ID/g, respectively. A longer blood retention would prevent fast binding of the bispecific tracers to their targets: PSMA in the LNCaP tumor xenografts and FAP in the HEK293T:hFAP tumor xenografts. This is evident by the observations that with no additional linker between the triazole ring and the FAP-targeting pharmacophore, [^68^Ga]Ga-AV01030 showed relatively higher tumor uptake and tumor-to-background (bone, muscle and blood) contrast ratios in both tested tumor models than those of [^68^Ga]Ga-AV01017 and [^68^Ga]Ga-AV01038.

It should be noted that evaluation by imaging and/or biodistribution studies in tumor-bearing mice as in this report is a common practice in the development of radiopharmaceuticals. However, the obtained high tumor-to-background contrast either from imaging or biodistribution data might not be observed in the clinic especially for ^68^Ga-labeled tracers. This is because compared with other positron emitters, ^68^Ga has a higher average positron energy (^68^Ga, 0.829 MeV; ^18^F, 0.250 MeV; ^64^Cu, 0.288 MeV; ^89^Zr, 0.396 MeV), leading to poorer spatial resolution and difficulty in the visualization of small lesions (a few millimeters in size). In addition, the tumor xenografts used in the imaging and biodistribution studies are often derived from (genetically-modified) cancer cell lines overexpressing the targeted cancer markers, which might not be representative of cancer lesions encountered in the clinic.

Directly comparing the performance of our bispecific tracers with those reported previously is difficult as different tumor models were used for evaluation: PSMA-expressing LNCaP tumors and FAP-expressing HEK293T:hFAP tumors used in this report; PSMA-expressing PC3-PIP tumors and FAP-expressing U87 tumors used by Boinapally et al. [32]; PSMA-expressing 22Rv1 tumors and FAP-expressing A549-FAP tumors used by Hu et al. [33]. However, comparing the pharmacokinetics of our tracers and those reported previously shows that our tracers had significantly higher blood retention at 1 h post injection. The longer blood retention of our tracers could be due to the increased lipophilicity [39], which could be a result of (1) the replacement of 2-Nal with a more lipophilic Ala(9-Anth) in the PSMA-targeting pharmacophore; (2) the deletion of the hydrophilic piperazine linker in the FAP-targeting pharmacophore of FAPI-04; and/or (3) the use of click reaction for coupling the FAP-targeting pharmacophore as this forms a relatively lipophilic triazole ring instead of a hydrophilic amide bond.

It should be noted that the same diagnostic information obtained from the use of a bispecific tracer could be obtained by two separate scans using its corresponding monospecific tracers. However, the use of a bispecific tracer would save the time and overall cost by reducing two procedures (hospital visit, tracer preparation, tracer injection, PET scan, scan reading, and report writing) to one, and reduce patients’ absorbed radiation dose from PET scanning. It should also be noted that compared to its corresponding monospecific tracers, a bispecific tracer would have a larger molecular size, and combining two different targeting vectors into a single molecule could potentially negatively impact both its pharmacokinetic properties and receptor binding affinity. However, with thorough investigations on the selection of targeting vectors, linkers, and potentially pharmacokinetic modifiers, it is likely to obtain an optimized bispecific tracer with higher tumor uptake and even higher tumor-to-background contrast ratios than both of its corresponding monospecific tracers. For example, the Chen group designed and evaluated gastrin-releasing peptide receptor (GRPR) and integrin α_v_β_3_ bispecific radiotracers for imaging prostate cancer [40]. GRPR is highly expressed by prostate cancer cells, whereas integrin α_v_β_3_ is expressed by tumor neovasculature. Their results showed that the GRPR/integrin α_v_β_3_ bispecific tracer ([^18^F]FB-BBN-RGD) achieved higher tumor uptake and tumor-to-background contrast ratios than both of its corresponding monospecific tracers ([^18^F]FB-BBN for GRPR and [^18^F]FB-RGD for integrin α_v_β_3_).

Further modifications are needed to improve the binding affinities, pharmacokinetics, and tumor uptake of ^68^Ga-labeled PSMA/FAP bispecific tracers by considering the linker’s length, lipophilicity, and the use of less lipophilic PSMA- and/or FAP-targeting pharmacophores. This can be accomplished by the use of less lipophilic linkers such as PEG linkers and well as piperazine-based linkers which have been shown to be important for maintaining good tumor uptake of FAP-targeting tracers [41]. Similarly, the use of potent and less lipophilic pharmacophores should also be investigated such as (1) replacing Ala(9-Anth) in the PSMA-targeting pharmacophore with 2-Nal, (2) replacing the quinoline motif in the FAP-targeting pharmacophore with a more hydrophilic pyridine motif, and/or (3) replacing the 2-pyrrolidinecarbonitrile motif in the FAP-targeting pharmacophore with a more hydrophilic pyrrolidin-2-ylboronic acid.

## 4. Materials and Methods

### 4.1. Synthesis of Bispecific PSMA/FAP-Targeting Ligands

Detailed information for the synthesis, purification, and characterizations of AV01017, AV01030, and AV01038, and their nonradioactive Ga-complexed standards and ^68^Ga-labeled analogs are provided in the supplemental information (Supplemental Tables S1–S3).

### 4.2. Cell Culture

The LNCaP cells obtained from ATCC (via Cedarlane, Burlington, ON, Canada) were cultured in RPMI 1640 medium supplemented with 10% FBS, penicillin (100 U/mL), and streptomycin (100 μg/mL) at 37 °C in a Panasonic Healthcare (Tokyo, Japan) MCO-19AIC humidified incubator containing 5% CO_2_. The cells were confirmed to be pathogen-free by the IMPACT Rodent Pathogen Test (IDEXX BioAnalytics). Cells were grown until 80–90% confluence and washed with sterile phosphate-buffered saline (PBS, pH 7.4) and collected after 1 min trypsinization. The cell concentration was counted in triplicate using a hemocytometer and a manual laboratory counter.

### 4.3. Cell Transfection

The HEK293T cells were obtained from ATCC. The FAP-expressing vector was constructed using Genome-CRISPRTM Human AAVS1 Safe Harbor Gene Knock-in Kits (GeneCopoeaiaTM) by inserting FAP-expressing gene into the AAVS1 vector. The cells were then transfected by the FAP-expressing vector following the EndofectinTM Transfection Reagent protocol. The cells underwent 3 serial dilutions and were sorted using fluorescence-activated cell sorting (FACS) to obtain FAP-expressing monoclonal colonies. HEK293T:hFAP cells were cultured in DMEM GlutaMAX™ medium supplemented with 10% FBS, penicillin (100 U/mL), and streptomycin (100 μg/mL) at 37 °C in a Panasonic Healthcare (Tokyo, Japan) MCO-19AIC humidified incubator containing 5% CO_2_. Cells were grown until 80–90% confluence and washed with sterile PBS (pH 7.4) and collected.

### 4.4. In Vitro PSMA Competition Binding Assay

The PSMA binding assays were conducted following previously published procedures using LNCaP cells and [^18^F]DCFPyL as the radioligand [34,42,43]. Data analyses of IC_50_ were performed using the nonlinear regression algorithm of GraphPad Prism 7 (San Diego, CA, USA) software.

### 4.5. In Vitro FAP Fluorescence Assay

The half maximal inhibitory concentration (IC_50_) values of the tested compounds for FAP were measured by in vitro enzymatic assay. The recombinant human FAP (Biolegend; 0.2 µg/mL, 50 µL) was added into a costar 96-well plate. PBS and varied concentrations (0.2 pM to 2 µM) of tested nonradioactive Ga-complexed standards were added to each well (in duplicate) containing the recombinant human FAP. After being incubated for 30 min at 37 °C, 50 µL of Suc-Gly-Pro-AMC (2 µM, Bachem) was added to each well. The fluorescent signals were acquired at 15, 30, 45, and 60 min using a FlexStation 3 Multi-Mode Microplate Reader with excitation at 380 nm and emission at 460 nm. The IC_50_(FAP) was calculated using a “nonlinear fit model” built-in model in GraphPad Prism 7.02 software

### 4.6. Ex Vivo Biodistribution and PET/CT Imaging Studies

Imaging and biodistribution studies were performed using male NOD.Cg-Rag1tm1Mom Il2rgtm1Wjl/SzJ (NRG) mice following previously published procedures [44,45,46]. The experiments were conducted according to the guidelines established by the Canadian Council on Animal Care and approved by Animal Ethics Committee of the University of British Columbia. The mice were briefly sedated by inhalation of 2.5% isoflurane in oxygen, and 100 µL LNCaP (2 × 10^5^ cells) or HEK293T:hFAP (8.5 × 10^6^ cells) cells were inoculated subcutaneously behind the left shoulder. When the tumor grew to 5–8 mm in diameter over 3–4 weeks and 4–5 weeks for HEK293T:hFAP and LNCaP tumors, respectively, the mice were used for PET/CT imaging and biodistribution studies.

PET/CT imaging experiments were carried out using a Siemens (Knoxville, TN) Inveon micro PET/CT scanner. Each tumor-bearing mouse was injected with ~4–6 MBq of ^68^Ga-labeled tracer through a lateral caudal tail vein under 2.5% isoflurane in oxygen anesthesia, followed by recovery and free roaming in its cage during the uptake period. At 50 min post injection, a 10-min CT scan was conducted first for localization and attenuation correction after segmentation for reconstructing the PET images, followed by a 10-min static PET imaging acquisition.

For biodistribution studies, the mice were injected with the radiotracer (~2–4 MBq) as described above. At 1 h post injection, the mice were euthanized by CO_2_ inhalation. Blood was withdrawn by cardiac puncture, and organs/tissues of interest were collected, weighed, and counted using a Perkin Elmer (Waltham, MA, USA) Wizard2 2480 automatic gamma counter.

### 4.7. Statistical Analysis

Data were analyzed with the GraphPad Prism, version 7.02, and Microsoft (Redmond, WA, USA) Excel software. One way ANOVA and multiple *t*-tests were performed for all organs in the biodistribution studies of [^68^Ga]Ga-AV01017, [^68^Ga]Ga-AV01030, [^68^Ga]Ga-AV01038, [^68^Ga]Ga-HTK03041, and [^68^Ga]Ga-FAPI-04 in LNCaP and HEK293T:hFAP tumor models. A statistically significant difference was considered present when the adjusted *p* value was less than 0.05 using the Holm–Sidak method.

## 5. Conclusions

Three novel ^68^Ga-labeled PSMA/FAP bispecific tracers were designed, synthesized, and confirmed to have the ability to bind both PSMA and FAP in vitro and in vivo. Compared with the monospecific tracers, the bispecific tracers have decreased binding affinities towards PSMA, but retain comparable binding affinities towards FAP. The bispecific tracers have lower tumor uptake values compared to the monospecific tracers, in which the tracer with a longer linker tends to have a lower tumor uptake. This might be caused by the longer blood retention of the bispecific tracers. Further modifications will be explored to improve the binding affinity, pharmacokinetics, and tumor uptake to generate promising PSMA/FAP bispecific radioligands for imaging and radioligand therapy of mCRPC.

## Data Availability

The data presented in this study are available in the Supplementary Materials.

## Supplementary Materials

The following supporting information can be downloaded at: https://www.mdpi.com/article/10.3390/molecules28031088/s1. Detailed synthetic procedures and results for the preparation of bispecific ligands and their ^nat^Ga/^68^Ga-complexed analogs; Table S1: HPLC purification conditions and MS characterizations of DOTA-conjugated precursors; Table S2: HPLC purification conditions and MS characterizations of nonradioactive Ga-complexed standards; Table S3: HPLC conditions for the purification and quality control of ^68^Ga-labeled tracers; Table S4: Biodistribution and uptake ratios of ^68^Ga-labeled PSMA/FAP bispecific tracers, HTK03041 and FAPI-04 in LNCaP tumor-bearing mice; Table S5: Biodistribution and uptake ratios of ^68^Ga-labeled PSMA/FAP bispecific tracers, HTK03041 and FAPI-04 in HEK293T:hFAP tumor-bearing mice [26,47,48,49,50].

## Sample Availability

Samples of the investigated compounds are available from A.V.
