# Supplementary material for: Synthesis and Preclinical Evaluation of Three Novel 68Ga-Labeled Bispecific PSMA/FAP-Targeting Tracers for Prostate Cancer Imaging
Source: Molecules. 2023 Jan 21;28(3):1088. doi: 10.3390/molecules28031088 (PMC9921851; doi:10.3390/molecules28031088)
Supplement: Supplementary file 1 [file molecules-28-01088-s001.zip › molecules-2151308-supplementary.pdf]

SUPPLEMENTAL MATERIALS

General Methods

Methyl 6-bromoquinoline-4-carboxylate, (*S*)-1-(2-aminoacetyl)-4,4-difluoropyrrolidine-2-carbonitrile 4-methylbenzenesulfonate, FAPI-04, HTK03041, Ga-HTK03041 and [^{68}Ga]Ga-HTK03041 were synthesized following literature procedures [1–4]. All other chemicals and solvents were obtained from commercial sources and used without further purification. DOTA-conjugated PSMA/FAP bispecific ligands were synthesized via SPPH with an AAPPTec (Louisville, KY) Endeavor 90 peptide synthesizer. Purification and quality control of DOTA-conjugated ligands and their ^{nat}Ga/^{68}Ga-complexed analogs were performed on Agilent (Santa Clara, CA) HPLC systems equipped with a model 1200 quaternary pump, a model 1200 UV absorbance detector (220 nm), and a Bioscan (Washington, DC) NaI scintillation detector. The HPLC columns used were a semi-preparative column (Luna C18, 5 µm, 250 × 10 mm) and an analytical column (Luna C18, 5 µm, 250 × 4.6 mm) purchased from Phenomenex (Torrance, CA). The collected HPLC eluates containing the desired peptides were lyophilized using a Labconco (Kansas City, MO) FreeZone 4.5 Plus freeze-drier. MS analyses were conducted using the Waters (Milford, MA) Acquity QDa mass spectrometer with the equipped 2489 UV/Vis detector and e2695 Separations module. C18 Sep-Pak cartridges (1 cm^3, 50 mg) were purchased from Waters (Milford, MA). ^{68}Ga was eluted from an ITM Medical Isotopes GmbH (Munich, Germany) generator, and purified according to the previously published procedures using a DGA resin column from Eichrom Technologies LLC (Lisle, IL) [5]. The radioactivity of ^{68}Ga-labeled tracers was measured using a Capintec (Ramsey, NJ) CRC®-25R/W dose calibrator and the radioactivity of mouse tissues collected from biodistribution studies were counted using a Perkin Elmer (Waltham, MA) Wizard2 2480 automatic gamma counter.

Synthesis of alkyne-containing FAP-targeting motifs 5, 9 and 13.

<u>Synthesis of methyl 6-hydroxyquinoline-4-carboxylate (**1**)</u>

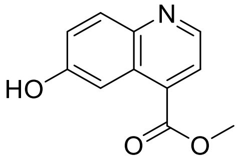

A solution of quininic acid (5.0 g, 24.6 mmol) in mixture of hydrogen bromide (48%, 100 mL) and ethanol (25 mL) was stirred for 72 h at 115 °C. The solution was evaporated under reduced pressure and the residue was dissolved in methanol (80 mL). The solution was added thionyl chloride (5 mL) and stirred at 60 °C. After stirred for 22 h, the solution was evaporated. The residue was dissolved in saturated sodium bicarbonate (80 mL), and extracted with water (100 mL × 3). The organic phases were combined, dried over anhydrous $MgSO_4$, filtered, and evaporated under reduced pressure to yield compound **1** as a yellow solid (2.32 g, 47% yield). MS (ESI) calculated for $C_{16}H_{15}NO_3$ 203.10, found $[M+H]^+$ 204.04 ^{1}H NMR (300 MHz, DMSO) δ 10.40 (s, 1H), 8.79 (d, *J* = 4.4 Hz, 1H), 7.98 (d, *J* = 6.4 Hz, 1H), 7.96 (s, 1H), 7.86 (d, *J* = 4.4 Hz, 1H), 7.39 (dd, *J* = 9.1, 2.7 Hz, 1H), 3.95 (s, 3H).

Synthesis of methyl 6-(pent-4-yn-1-yloxy)quinoline-4-carboxylate (**2**)

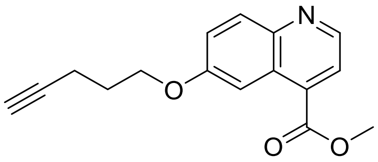

To a solution of methyl 6-hydroxyquinoline-4-carboxylate **1** (1.73 g, 8.5 mmol), 4-pentyn-1-ol (7.57 g, 9.0 mmol) and triphenylphosphine (2.36 g, 9.0 mmol) in THF (50 mL) cooled in an ice/water bath was added diisopropyl azodicarboxylate (DIAD) (1.82 g, 9 mmol) dropwise. After stirring for 20 h, the resulting solution was evaporated and purified using silica gel flash column chromatography eluted with 3:7 (v/v) ethyl acetate/hexanes. The collected product eluate fractions were combined, evaporated and dried under reduced pressure to yield **2** as a white powder (2.28 g, 100% yield). MS (ESI) calculated for $C_{16}H_{15}NO_3$ 269.11, found $[M+H]^+$ 270.08. ^{1}H NMR (300 MHz, DMSO) δ 8.89 (d, J = 4.5 Hz, 2H), 8.12 – 8.00 (m, 2H), 7.95 (d, J = 4.5 Hz, 1H), 7.53 (dd, J = 9.2, 2.8 Hz, 1H), 4.20 (t, J = 6.2 Hz, 2H), 3.98 (s, 3H), 2.40 (td, J = 7.1, 2.7 Hz, 2H), 1.99 (p, J = 6.6 Hz, 2H).

Synthesis of 6-(pent-4-yn-1-yloxy)quinoline-4-carboxylic acid (**3**)

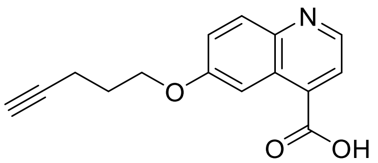

A solution of compound **2** (2.28 g, 8.5 mmol) and sodium hydroxide (2.4 g, 61 mmol) in a mixture of methanol (25 mL) and water (20 mL) was stirred for 16 h. The solution was evaporated under reduced pressure and the residue was dissolved in water (80 mL). The aqueous solution was acidified with concentrated HCl to pH 3. The resulting white precipitates were collected by filtration and dried under reduced pressure to yield compound **3** as a white powder (1.61 g, 74% yield). MS (ESI) calculated for $C_{15}H_{13}NO_3$ 255.09, found $[M+H]^+$ 256.08. ^{1}H NMR (300 MHz, DMSO) δ 8.87 (d, J = 4.4 Hz, 1H), 8.17 (d, J = 2.8 Hz, 1H), 8.03 (d, J = 9.2 Hz, 1H), 7.93 (d, J = 4.4 Hz, 1H), 7.50 (dd, J = 9.2, 2.8 Hz, 1H), 4.18 (t, J = 6.2 Hz, 2H), 2.82 (t, J = 2.6 Hz, 1H), 2.40 (td, J = 7.1, 2.7 Hz, 2H), 1.99 (p, J = 6.6 Hz, 2H).

Synthesis of 2,3,5,6-tetrafluorophenyl 6-(pent-4-yn-1-yloxy)quinoline-4-carboxylate (**4**)

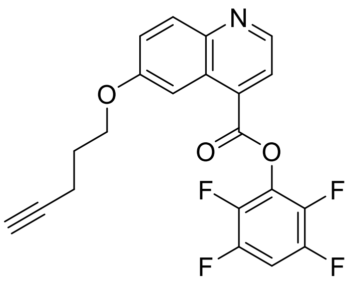

To a solution of compound **3** (1.56 g, 6.1 mmol) and 2,3,5,6-tetrafluorophenol (1.22 g, 7.3 mmol) in DMF (25 mL) cooled in an ice/water bath was added *N*,*N'*-dicyclohexylcarbodiimide (DCC, 1.26 g, 6.1 mmol) in DMF (25 mL) dropwise. After stirring for 72 h at room temperature, the resulting solution was filtered through celite and the organic solvent was evaporated under reduced pressure. The residue was purified by silica gel flash column chromatography eluted with 1:3 (v/v) ethyl acetate/hexanes. The collected product eluate fractions were combined, evaporated and dried under reduced pressure to yield **4** as a light yellow solid (1.77 g, 72% yield). MS (ESI) calculated for $C_{21}H_{13}F_4NO_3$ 403.08, found $[M+H]^+$ 404.13. ^{1}H NMR (300 MHz, DMSO) δ 8.86 (d, *J* = 4.4 Hz, 1H), 8.17 (d, *J* = 2.8 Hz, 1H), 8.02 (d, *J* = 9.2 Hz, 1H), 7.92 (d, *J* = 4.4 Hz, 1H), 7.50 (dd, *J* = 9.2, 2.8 Hz, 1H), 7.19 (tt, *J* = 10.9, 7.2 Hz, 1H), 4.18 (t, *J* = 6.2 Hz, 2H), 2.82 (t, *J* = 2.6 Hz, 1H), 2.40 (td, *J* = 7.1, 2.7 Hz, 2H), 1.99 (p, *J* = 6.6 Hz, 2H).

Synthesis of (*S*)-*N*-(2-(2-cyano-4,4-difluoropyrrolidin-1-yl)-2-oxoethyl)-6-(pent-4-yn-1-yloxy)quinoline-4-carboxamide (**5**)

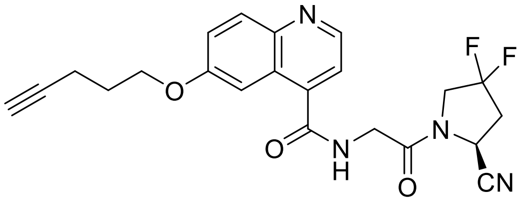

A solution of compound **4** (403 mg, 1.0 mmol), (*S*)-1-(2-aminoacetyl)-4,4-difluoropyrrolidine-2-carbonitrile 4-methylbenzenesulfonate (1.0 mmol) and triethylamine (202 g, 2 mmol) in a mixture of CH_2Cl_2 (8 mL) and CH_3CN (8 mL) was stirred at 50°C for 23 h. The resulting solution was evaporated under reduced procedure and the residue was purified through silica gel flash column chromatography eluted with 1:5 (v/v) methanol/ethyl acetate. The collected product eluate fractions were combined, evaporated and dried under reduced pressure to yield **5** as a light yellow solid (415 mg, 97% yield). MS (ESI) calculated for $C_{22}H_{20}F_2N_4O_3$ 426.15, found $[M+H]^+$ 427.22. ^{1}H NMR (300 MHz, DMSO) δ 9.10 (t, *J* = 6.0 Hz, 1H), 8.81 (d, *J* = 4.3 Hz, 1H), 7.99 (d, *J* = 9.2 Hz, 1H), 7.51 (d, *J* = 4.3 Hz, 1H), 7.47 (dd, *J* = 9.2, 2.7 Hz, 1H), 5.14 (dd, *J* = 9.1, 3.0 Hz, 1H), 4.50 – 4.02 (m, 6H), 2.80 (t, *J* = 2.6 Hz, 1H), 2.38 (td, *J* = 7.1, 2.7 Hz, 2H), 1.98 (m, *J* = 6.7 Hz, 2H).

Synthesis of methyl 6-(2-(trimethylsilyl)ethynyl)quinoline-4-carboxylate (**6**)

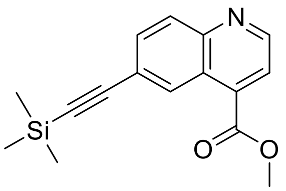

A solution of methyl 6-bromoquinoline-4-carboxylate (1.33 g, 5.0 mmol), tetrakis(triphenylphosphine)palladium(0) (175 mg, 0.25 mmol), copper (I) iodide (95 mg, 0.50 mmol) and ethynyltrimethylsilane (982 mg, 10 mmol) in TEA (12 mL) was stirred at 90 °C for 18 h. The solution was diluted with water (100 mL) and then filtered through celite. The filtered aqueous solution was extracted with ethyl acetate (100 mL × 3). The organic phases were

combined, dried over anhydrous $MgSO_4$, filtered, and evaporated under reduced pressure. The residue was purified with silica gel flash column chromatography eluted with 1:1 (v/v) diethyl ether/hexanes. The product eluate fractions were combined and evaporated under reduced pressure to yield **6** as a yellow oil (880 mg, 62% yield). MS (ESI) calculated for $C_{16}H_{17}NO_2Si$ 283.10, found $[M+H]^+$ 284.07. ^1H NMR (300 MHz, DMSO) δ 9.09 (d, *J* = 4.4 Hz, 1H), 8.74 (d, *J* = 1.8 Hz, 1H), 8.11 (d, *J* = 8.7 Hz, 1H), 8.00 (d, *J* = 4.4 Hz, 1H), 7.84 (dd, *J* = 8.7, 1.9 Hz, 1H), 3.99 (s, 3H).

Synthesis of 6-ethynylquinoline-4-carboxylic acid (**7**)

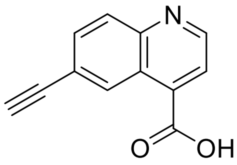

A solution of compound **6** (880 mg, 3.1 mmol) and lithium hydroxide (30 mmol) in a mixture of water (25 mL) and methanol (25 mL) was stirred at 60 °C for 3 h. After evaporation, the residue was dissolved in water (40 mL), and washed with diethyl ether (80 mL). The aqueous phase was then acidified using concentrated HCl to pH 3. The resulting precipitates were collected and dried under reduced pressure to yield **7** as a yellow powder (509 mg, 83% yield). MS (ESI) calculated for $C_{12}H_7NO_3$ 197.05, found $[M+H]^+$ 198.06. ^1H NMR (300 MHz, DMSO) δ 9.15 (d, *J* = 4.3 Hz, 1H), 8.98 (d, *J* = 1.8 Hz, 1H), 8.20 (d, *J* = 8.7 Hz, 1H), 8.07 (d, *J* = 4.4 Hz, 1H), 7.93 (dd, *J* = 8.7, 1.6 Hz, 1H), 4.51 (s, 1H).

Synthesis of 2,3,5,6-tetrafluorophenyl 6-ethynylquinoline-4-carboxylate (**8**)

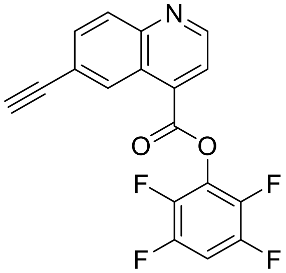

To a solution of compound **7** (495 mg, 2.5 mmol) and 2,3,5,6-tetrafluorophenol (540 mg, 3.2 mmol) in DMF (20 mL) cooled in an ice/water bath was added dropwise a solution of DCC (660 mg, 3.2 mmol) in DMF (10 mL). The resulting solution was stirred for 21 h at room temperature, filtered, and the filtrate was evaporated under reduced pressure. The residue was purified using silica gel flash column chromatography eluted with 2:3 (v/v) diethyl ether/hexanes. The product eluate fractions were combined, evaporated and dried under reduced pressure to yield **8** as a light yellow solid (615 mg, 71% yield). MS (ESI) calculated for $C_{18}H_7F_4NO_3$ 345.04, found $[M+H]^+$ 346.03. ^1H NMR (300 MHz, DMSO) δ 9.07 (d, *J* = 4.4 Hz, 1H), 8.89 (d, *J* = 1.8 Hz, 1H), 8.11 (d, *J* = 8.7 Hz, 1H), 8.00 (d, *J* = 4.4 Hz, 1H), 7.85 (dd, *J* = 8.7, 1.9 Hz, 1H), 7.30 – 7.11 (m, 1H), 4.42 (s, 1H).

Synthesis of (*S*)-*N*-(2-(2-cyano-4,4-difluoropyrrolidin-1-yl)-2-oxoethyl)-6-ethynylquinoline-4-carboxamide (**9**)

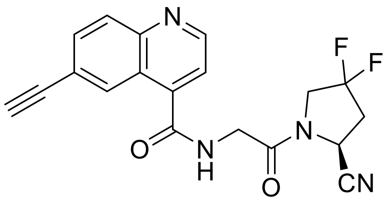

A solution of compound **8** (605 mg, 1.8 mmol), (*S*)-1-(2-aminoacetyl)-4,4-difluoropyrrolidine-2-carbonitrile 4-methylbenzenesulfonate (2.1 g, 2.1 mmol) and TEA (607 mg, 6.0 mmol) in a mixture of CH_2Cl_2 (15 mL) and CH_3CN (15 mL) was stirred at 50 °C for 19 h. The solution was then evaporated and purified through silica gel flash column chromatography eluted with 1:9 (v/v) methanol/ethyl acetate. The product eluate fractions were combined, evaporated and dried under reduced pressure to yield **9** as a white solid (644 mg, 100% yield). MS (ESI) calculated for $C_{19}H_{14}F_2N_4O_2$ 368.11, found $[M+H]^+$ 369.20. ^1H NMR (300 MHz, DMSO) δ 9.20 (t, *J* = 5.9 Hz, 1H), 9.03 (d, *J* = 4.3 Hz, 1H), 8.46 (d, *J* = 1.8 Hz, 1H), 8.09 (d, *J* = 8.7 Hz, 1H), 7.84 (dd, *J* = 8.7, 1.9 Hz, 1H), 7.63 (d, *J* = 4.3 Hz, 1H), 5.19 (dd, *J* = 9.1, 2.8 Hz, 1H), 4.35 – 4.05 (m, 2H), 3.05 – 2.78 (m, 2H).

Synthesis of methyl 6-(prop-2-yn-1-yloxy)quinoline-4-carboxylate (**10**)

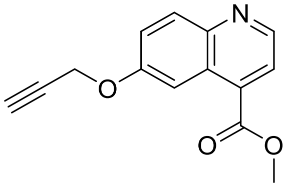

To a solution of methyl 6-hydroxyquinoline-4-carboxylate **1** (1.13 g, 5.6 mmol), 2-propyn-1-ol (348 mg, 6.2 mmol) and triphenylphosphine (1.63 g, 6.2 mmol) in THF (30 mL) cooled in an ice/water bath was added dropwise a solution of DIAD (1.25 g, 6.2 mmol) in THF (10 mL). The resulting solution was stirred for 20 h at room temperature. The solution was then evaporated and purified through silica gel flash column chromatography eluted with 6:4 (v/v) ethyl acetate:hexanes. The product eluate fractions were combined, evaporated and dried under reduced pressure to yield **10** as a white powder (1.35 g, 100% yield). MS (ESI) calculated for $C_{14}H_{11}NO_3$ 241.07, found $[M+H]^+$ 242.05. ^1H NMR (300 MHz, DMSO) δ 8.92 (d, *J* = 4.5 Hz, 1H), 8.19 (d, *J* = 2.8 Hz, 1H), 8.08 (d, *J* = 9.2 Hz, 1H), 7.95 (d, *J* = 4.5 Hz, 1H), 7.56 (dd, *J* = 9.2, 2.8 Hz, 1H), 4.97 (d, *J* = 2.4 Hz, 2H), 3.99 (s, 3H), 3.64 (t, *J* = 2.4 Hz, 1H).

Synthesis of 6-(prop-2-yn-1-yloxy)quinoline-4-carboxylic acid (**11**)

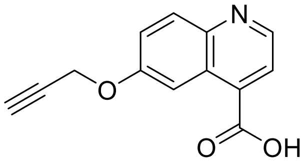

A solution of compound **10** (1.35 g, 5.6 mmol) and sodium hydroxide (2.52 g, 63 mmol) in a mixture of water (20 mL) and methanol (25 mL) was stirred for 17 h. After evaporation, the residue was dissolved in water and the resulting solution was acidified with concentrated HCl to pH 3. The resulting white precipitates were collected by filtration and dried under reduced pressure to yield **11** as a yellow solid (927 mg, 73% yield). MS (ESI) calculated for $C_{13}H_9NO_3$ 227.06, found [M+H]+ 228.09. ^{1}H NMR (300 MHz, DMSO) δ 8.89 (d, *J* = 4.4 Hz, 1H), 8.27 (d, *J* = 2.9 Hz, 1H), 8.06 (d, *J* = 9.2 Hz, 1H), 7.93 (d, *J* = 4.4 Hz, 1H), 7.54 (dd, *J* = 9.2, 2.9 Hz, 1H), 4.95 (d, *J* = 2.4 Hz, 2H), 3.62 (t, *J* = 2.4 Hz, 1H).

Synthesis of 2,3,5,6-tetrafluorophenyl 6-(prop-2-yn-1-yloxy)quinoline-4-carboxylate (**12**)

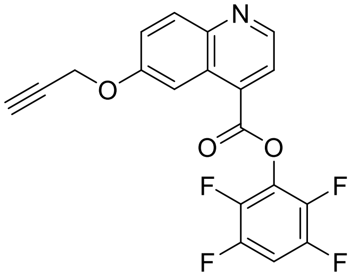

To a solution of compound **11** (900 mg, 4.0 mmol) and 2,3,5,6-tetrafluorophenol (830 mg, 5.0 mmol) in DMF (20 mL) cooled in an ice/water bath was added dropwise a solution of DCC (825 mg, 4.0 mmol) in DMF (20 mL). The resulting solution was stirred for 22 h and then filtered. The filtrate was evaluated under reduced pressure and the residue was purified through silica gel flash column chromatography eluted with 4:6 (v/v) diethyl ether/hexanes. The product eluate fractions were combined, evaporated and dried under reduced pressure to yield **12** as a yellow solid (1.02 g, 68% yield). MS (ESI) calculated for $C_{19}H_9F_4NO_3$ 375.05, found $[M+H]^+$ 376.06. ^{1}H NMR (300 MHz, DMSO) δ 8.89 (d, *J* = 4.4 Hz, 1H), 8.28 (d, *J* = 2.8 Hz, 1H), 8.06 (d, *J* = 9.2 Hz, 1H), 7.94 (d, *J* = 4.4 Hz, 1H), 7.54 (dd, *J* = 9.2, 2.9 Hz, 1H), 7.22 (q, *J* = 8.4 Hz, 1H), 4.95 (s, 2H), 3.62 (d, *J* = 4.5 Hz, 1H).

Synthesis of (*S*)-*N*-(2-(2-cyano-4,4-difluoropyrrolidin-1-yl)-2-oxoethyl)-6-(prop-2-yn-1-yloxy)quinoline-4-carboxamide (**13**)

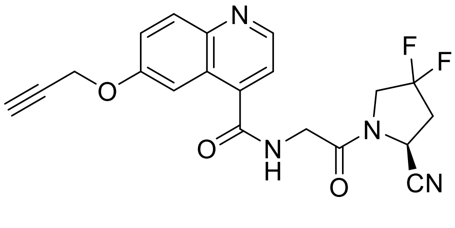

A solution of compound **12** (500 mg, 1.3 mmol), (*S*)-1-(2-aminoacetyl)-4,4-difluoropyrrolidine-2-carbonitrile 4-methylbenzenesulfonate (481 mg, 1.3 mmol) and TEA (3.0 mmol, 304 mg) in a mixture of CH_2Cl_2 (8 mL) and CH_3CN (8 mL) was stirred at 50 °C for 22 h. The solution was then evaporated under reduced pressure and the residue was purified using silica gel flash column chromatography eluted with 1:5 (v/v) methanol/ethyl acetate. The product eluate fractions were collected, combined, evaporated and dried under reduced pressure to yield **13** as a white solid (528 mg, 100% yield). MS (ESI) calculated for $C_{20}H_{16}F_2N_4O_3$ 398.12, found $[M+H]^+$

399.15. ^{1}H NMR (300 MHz, DMSO) δ 9.09 (t, *J* = 6.0 Hz, 1H), 8.83 (dd, *J* = 10.2, 4.4 Hz, 1H), 8.02 (t, *J* = 9.8 Hz, 1H), 7.88 (d, *J* = 2.9 Hz, 1H), 7.59 – 7.45 (m, 1H), 5.16 (dd, *J* = 9.2, 2.9 Hz, 1H), 4.41 – 4.02 (m, 4H), 3.06 – 2.74 (m, 3H).

Synthesis of DOTA-conjugated Ligands

AV01017, AV01030, and AV01038 were synthesized on solid phase using Fmoc peptide chemistry. Fmoc-Lys(ivDde)-Wang resin (0.05 mmol, 0.081 mg) was treated with 20% piperidine in DMF to remove the Fmoc protecting group. The isocyanate derivative (3 eq.) of Glu(tBu)-OtBu was synthesized following previously published procedures [6] and was added to the lysine-immobilized resin, with *N,N*-diisopropylethylamine (DIEA, 2 eq.) as the base and reacted for 16 h to form the Lys-urea-Glu moiety. The ivDde-protecting group was then removed with 2% hydrazine in DMF (5 × 5 min). Fmoc-Ala(9-Anth)-OH (3 eq.), Fmoc-tranexamic acid (3 eq.), Fmoc-Gly-OH (3 eq.), and Fmoc-Lys(ivDde)-OH (3 eq.) were pre-activated with HATU (3 eq.) and DIEA (3 eq.) before being sequentially coupled to the resin. Following the removal of Fmoc protecting group, azidoacetic acid (2 eq.) was pre-activated with DIC (3 eq.) and DIEA (3 eq.) and coupled to the α-amino group of lysine. A click reaction was then performed between the alkyne-containing FAP-targeting moiety (compound **5**, **9** or **13**, 2 eq.) and the azido group on resin in the presence of $CuSO_4$ (1 M, 10 µL), tris(benzyltriazolylmethyl)amine (TBTA, 3eq.) and ascorbic acid (1 M, 100 µL) for 48 h. Finally, the ivDde-protecting group on lysine was removed as described above, and the free amino group was coupled with DOTA-tris(*t*-butyl)ester activated with HATU (3 eq.) and DIEA (7 eq.).

The bispecific ligands were deprotected and simultaneously cleaved from the resin with a mixture of trifluoroacetic acid (TFA, 95%), triisopropylsilane (TIS 2.5%) and water (2.5%) for 4 h at room temperature. The cleaved ligands were filtered and then precipitated in cold diethyl ether. The crude ligands were pelleted by centrifugation and purified with HPLC (semi-preparative column; flow rate: 4.5 mL/min). The eluates containing the desired products were collected and lyophilized. The HPLC conditions, retention times, isolated yields and MS confirmations of DOTA-conjugated bispecific are provided in the Supplemental Table 1.

Synthesis of Nonradioactive Ga-complexed Standards

The nonradioactive Ga-complexed standards were prepared by reacting the DOTA-conjugated precursors with $GaCl_3$ (5 eq.) in NaOAc buffer (0.1 M, 500 µL, pH 4.2 – 4.5) at 80 °C for 15 min. The reaction mixture was then purified via HPLC (semi-preparative column, flow rate: 4.5 mL/min). The HPLC eluates containing the desired products were collected and lyophilized. The HPLC conditions, retention times, isolated yields and MS confirmations of these nonradioactive Ga-complexed standards are provided in the Supplemental Table 2.

Synthesis of ^{68}Ga-labeled Compounds

The radiolabeling experiments were performed according to previously published procedures [6, 7]. Purified $^{68}GaCl_3$ in 0.5 mL water was added to a 4-mL glass vial preloaded with 0.7 mL of HEPES buffer (2 M, pH 5.0) and 10 µL precursor solution (1 mM). The radiolabeling reaction was carried out under microwave heating for 1 min, followed by purification using HPLC semi-preparative column. The eluate fraction containing the radiolabeled product was collected,

diluted with water (50 mL), and passed through a C18 Sep-Pak cartridge that was pre-washed with ethanol (10 mL) and water (10 mL). The C18 Sep-Pak cartridge was washed with water (10 mL), and the ^{68}Ga-labeled product was eluted off the cartridge with ethanol (0.4 mL). The eluted product was diluted with PBS for imaging and biodistribution studies. Quality control was performed using the analytical column. The HPLC conditions and retention times are provided in the Supplemental Table 3. The tracers were obtained in 43 – 60% decay-corrected radiochemical yields with > 74 GBq/μmol molar activity and > 99% radiochemical purity.

Table S1: HPLC purification conditions and MS characterizations of DOTA-conjugated precursors.

Compound name	HPLC conditions	Retention time (min)	Yield (%)	Calculated mass (m/z)	Found (m/z)
AV01017	29% CH_3CN and 0.1% TFA in H_2O	8.3	1.5	$[M+2H]^{2+}$ 893.9	$[M+2H]^{2+}$ 893.6
AV01030	29% CH_3CN and 0.1% TFA in H_2O	11.3	4.0	$[M+2H]^{2+}$ 864.9	$[M+2H]^{2+}$ 865.4
AV01038	28% CH_3CN and 0.1% TFA in H_2O	13.5	1.3	$[M+2H]^{2+}$ 879.9	$[M+2H]^{2+}$ 880.2

Table S2: HPLC purification conditions and MS characterizations of nonradioactive Ga-complexed standards.

Compound name	HPLC conditions	Retention time (min)	Yield (%)	Calculated mass (m/z)	Found (m/z)
Ga-AV01017	30% CH_3CN and 0.1% TFA in H_2O	7.5	24.8	$[M+2H]^{2+}$ 928.4	$[M+2H]^{2+}$ 927.7
Ga-AV01030	30% CH_3CN and 0.1% TFA in H_2O	11.5	24.5	$[M+2H]^{2+}$ 899.3	$[M+2H]^{2+}$ 899.8
Ga-AV01038	28% CH_3CN and 0.1% TFA in H_2O	15.9	88.9	$[M+2H]^{2+}$ 914.4	$[M+2H]^{2+}$ 913.5
Ga-FAPI-04	10% CH_3CN and 0.1% TFA in H_2O	10.5	8.7	$[M+2H]^{2+}$ 471.65	$[M+2H]^{2+}$ 470.29

Table S3: HPLC conditions for the purification and quality control of ^{68}Ga-labeled tracers. FA: formic acid.

Compound	HPLC conditions		Retention time (min)
[^{68}Ga]Ga-AV01017	Semi-Prep	29% CH_3CN and 0.1% FA in H_2O; flow rate 4.5 mL/min	17.0
	QC	31% CH_3CN and 0.1% FA in H_2O; flow rate 2 mL/min	5.1
[^{68}Ga]Ga-AV01030	Semi-Prep	29% CH_3CN and 0.1% FA in H_2O; flow rate 4.5 mL/min	16.2
	QC	31% CH_3CN and 0.1% FA in H_2O; flow rate 2 mL/min	6.0
[^{68}Ga]Ga-AV01038	Semi-Prep	31% CH_3CN and 0.1% FA in H_2O; flow rate 4.5 mL/min	10.3
	QC	30% CH_3CN and 0.1% FA in H_2O; flow rate 2 mL/min	5.8
[^{68}Ga]Ga-FAPI-04	Semi-Prep	11% CH_3CN and 0.1% TFA in H_2O; flow rate 4.5 mL/min	24.5
	QC	16% CH_3CN and 0.1% TFA in H_2O; flow rate 2 mL/min	5.2

Table S4: Biodistribution (mean ± SD, n = 4) and uptake ratios of ^{68}Ga-labeled PSMA/FAP bispecific tracers, HTK03041, and FAPI-04 in LNCaP tumor-bearing mice.

Tissue (%ID/g)	[^{68}Ga]Ga-AV01017	[^{68}Ga]Ga-AV01030	[^{68}Ga]Ga-AV01038	[^{68}Ga]Ga-HTK03041*	[^{68}Ga]Ga-FAPI-04
	1 h	1 h	1 h	1 h	1 h
Blood	9.24 ± 1.55	5.75 ± 0.59	7.07 ± 0.31	1.43 ± 0.30	2.16 ± 0.33
Fat	1.01 ± 0.48	0.68 ± 0.12	1.01 ± 0.28	2.06 ± 0.59	0.55 ± 0.47
Seminal vesicle	1.12 ± 0.21	1.04 ± 0.13	1.51 ± 0.08	-	3.72 ± 5.09
Testes	0.69 ± 0.06	0.70 ± 0.08	1.04 ± 0.10	1.34 ± 0.22	0.42 ± 0.04
Small Intestine	2.47 ± 0.48	1.58 ± 0.13	2.12 ± 0.24	1.14 ± 0.18	0.74 ± 0.12
Large Intestine	1.76 ± 0.21	1.32 ± 0.29	1.65 ± 0.31	-	0.73 ± 0.09
Stomach	0.64 ± 0.11	0.44 ± 0.07	0.64 ± 0.11	0.41 ± 0.11	0.26 ± 0.04
Spleen	3.73 ± 1.19	6.50 ± 1.37	6.77 ± 4.86	8.95 ± 3.22	0.67 ± 0.11
Liver	2.24 ± 0.34	1.43 ± 0.26	2.06 ± 0.13	1.38 ± 0.25	0.76 ± 0.08
Pancreas	5.38 ± 1.29	3.63 ± 0.29	3.91 ± 0.20	1.47 ± 0.16	0.99 ± 0.25
Adrenal glands	6.24 ± 2.31	3.13 ± 1.25	3.69 ± 2.54	-	2.16 ± 1.08
Kidneys	30.9 ± 6.89	44.0 ± 2.42	40.5 ± 8.26	170 ± 26.4	2.36 ± 0.38
Lungs	3.76 ± 0.66	2.93 ± 0.28	3.74 ± 0.78	4.32 ± 4.32	1.23 ± 0.07
Heart	4.04 ± 0.28	2.41 ± 0.13	3.26 ± 0.24	1.82 ± 0.62	0.70 ± 0.04
LNCaP tumor	4.38 ± 0.55	5.17 ± 0.51	4.25 ± 0.86	23.1 ± 6.11	3.15 ± 1.43
Muscle	1.91 ± 0.43	1.59 ± 0.14	1.68 ± 0.21	0.75 ± 0.09	1.04 ± 0.20
Bone	4.05 ± 0.86	4.48 ± 0.59	4.64 ± 1.70	1.29 ± 0.45	4.27 ± 0.74
Brain	0.18 ± 0.01	0.12 ± 0.01	0.15 ± 0.02	0.10 ± 0.05	0.06 ± 0.00
Thyroid	12.7 ± 2.72	8.33 ± 0.41	11.1 ± 1.62	2.48 ± 0.44	3.62 ± 1.01
Salivary glands	5.98 ± 4.00	4.76 ± 1.79	6.41 ± 1.19	-	1.36 ± 0.92
Lacrimal glands	0.13 ± 0.27	0.44 ± 0.68	0.06 ± 0.13	-	0.06 ± 0.07
Tumor/bone	1.10 ± 0.15	1.17 ± 0.26	0.97 ± 0.30	20.1 ± 9.48	0.51 ± 0.28
Tumor/muscle	2.35 ± 0.35	3.25 ± 0.39	2.52 ± 0.33	31.6 ± 12.1	2.30 ± 0.79
Tumor/blood	0.48 ± 0.11	0.89 ± 0.03	0.60 ± 0.13	17.3 ± 7.24	1.04 ± 0.42
Tumor/kidney	0.14 ± 0.03	0.11 ± 0.00	0.10 ± 0.02	0.14 ± 0.04	0.90 ± 0.28

*The biodistribution of [^{68}Ga]Ga-HTK03041 has been reported previously [4].

Table S5: Biodistribution (mean ± SD, n = 4) and uptake ratios of ^{68}Ga-labeled PSMA/FAP bispecific tracers, HTK03041, and FAPI-04 in HEK:FAP tumor-bearing mice.

Tissue (%ID/g)	[^{68}Ga]Ga-AV01017	[^{68}Ga]Ga-AV01030	[^{68}Ga]Ga-AV01038	[^{68}Ga]Ga-HTK03041	[^{68}Ga]Ga-FAPI-04
	1 h	1 h	1 h	1 h	1 h
Blood	11.9 ± 3.37	5.68 ± 0.72	7.45 ± 1.70	0.71 ± 0.07	1.07 ± 0.08
Fat	0.61 ± 0.13	0.63 ± 0.20	0.53 ± 0.10	0.55 ± 0.12	0.10 ± 0.01
Seminal vesicle	1.15 ± 0.33	0.64 ± 0.15	1.15 ± 0.69	0.32 ± 0.03	0.15 ± 0.04
Testes	1.15 ± 0.86	0.66 ± 0.08	0.74 ± 0.09	0.60 ± 0.41	0.28 ± 0.04
Small Intestine	2.14 ± 0.65	1.23 ± 0.16	1.70 ± 0.19	0.71 ± 0.01	0.36 ± 0.08
Large Intestine	1.44 ± 0.20	1.15 ± 0.10	0.99 ± 0.17	0.43 ± 0.04	-
Stomach	0.63 ± 0.08	0.42 ± 0.04	0.60 ± 0.18	0.22 ± 0.04	0.07 ± 0.01
Spleen	3.63 ± 3.60	7.41 ± 1.75	5.47 ± 1.03	8.09 ± 2.37	0.56 ± 0.11
Liver	3.40 ± 0.64	1.51 ± 0.33	2.16 ± 0.33	0.72 ± 0.06	0.36 ± 0.02
Pancreas	3.80 ± 0.25	3.26 ± 0.82	3.82 ± 1.29	0.71 ± 0.07	0.37 ± 0.05
Adrenal glands	4.06 ± 0.27	3.15 ± 0.39	6.14 ± 3.79	4.18 ± 0.48	1.07 ± 0.32
Kidneys	19.3 ± 2.25	30.0 ± 8.71	25.0 ± 1.76	68.4 ± 19.2	1.83 ± 0.16
Lungs	5.50 ± 1.26	3.02 ± 0.25	3.83 ± 0.55	2.43 ± 0.08	0.74 ± 0.11
Heart	4.63 ± 0.42	2.20 ± 0.46	2.69 ± 0.22	0.98 ± 0.07	0.34 ± 0.04
HEK293T:hFAP tumor	2.99 ± 0.37	3.69 ± 0.81	3.64 ± 0.83	0.62 ± 0.19	12.5 ± 2.00
Muscle	1.63 ± 0.06	1.78 ± 0.58	1.23 ± 0.11	0.47 ± 0.03	0.67 ± 0.05
Bone	5.26 ± 0.32	3.59 ± 0.44	3.03 ± 1.12	0.42 ± 0.11	3.36 ± 1.09
Brain	0.21 ± 0.07	0.12 ± 0.02	0.13 ± 0.02	0.03 ± 0.00	0.04 ± 0.00
Thyroid	10.7 ± 2.15	7.70 ± 1.08	6.51 ± 1.07	1.38 ± 0.10	-
Salivary glands	6.43 ± 1.03	7.01 ± 2.25	4.02 ± 0.69	2.29 ± 0.16	-
Lacrimal gland	1.34 ± 0.50	0.20 ± 0.24	0.22 ± 0.16	0.25 ± 0.33	-
Tumor/bone	0.44 ± 0.12	1.04 ± 0.31	1.17 ± 0.40	1.39 ± 0.58	3.93 ± 1.16
Tumor/muscle	1.25 ± 0.17	2.12 ± 0.30	2.21 ± 0.17	1.22 ± 0.73	18.8 ± 4.09
Tumor/blood	0.18 ± 0.08	0.65 ± 0.15	0.49 ± 0.37	0.83 ± 0.54	11.7 ± 2.04
Tumor/kidney	0.11 ± 0.03	0.64 ± 0.65	0.11 ± 0.02	0.01 ± 0.01	6.85 ± 1.33

References

1 Jansen, K.; Heirbaut, L.; Verkerk, R.; Cheng, J. D.; Joossens, J.; Cos, P.; Maes, L.; Lambeir, A.-M.; De Meester, I.; Augustyns, K.; et al. Extended Structure–Activity Relationship and Pharmacokinetic Investigation of (4-Quinolinoyl)Glycyl-2-Cyanopyrrolidine Inhibitors of Fibroblast Activation Protein (FAP). *J. Med. Chem.* **2014**, *57*, 3053–3074.

2 North, A. J.; Karas, J. A.; Ma, M. T.; Blower, P. J.; Ackermann, U.; White, J. M.; Donnelly, P. S. Rhenium and Technetium-Oxo Complexes with Thioamide Derivatives of Pyridylhydrazine Bifunctional Chelators Conjugated to the Tumour Targeting Peptides Octreotate and Cyclic-RGDfK. *Inorg. Chem.* **2017**, *56*, 9725–9741.

3 Lindner, T.; Loktev, A.; Altmann, A.; Giesel, F.; Kratochwil, C.; Debus, J.; Jäger, D.; Mier, W.; Haberkorn, U. Development of Quinoline-Based Theranostic Ligands for the Targeting of Fibroblast Activation Protein. *J. Nucl. Med.* **2018**, *59*, 1415–1422.

4 Kuo, H.-T.; Pan, J.; Zhang, Z.; Lau, J.; Merkens, H.; Zhang, C.; Colpo, N.; Lin, K.-S.; Bénard, F. Effects of Linker Modification on Tumor-to-Kidney Contrast of ^{68}Ga-Labeled PSMA-Targeted Imaging Probes. *Mol. Pharmaceutics* **2018**, *15*, 3502–3511.

5 Lin, K.-S.; Pan, J.; Amouroux, G.; Turashvili, G.; Mesak, F.; Hundal-Jabal, N.; Pourghiasian, M.; Lau, J.; Jenni, S.; Aparicio, S.; et al. In Vivo Radioimaging of Bradykinin Receptor B1, a Widely Overexpressed Molecule in Human Cancer. *Cancer Res.* **2015**, *75*, 387–393.

6 Zhang, C.; Zhang, Z.; Lin, K.-S.; Pan, J.; Dude, I.; Hundal-Jabal, N.; Colpo, N.; Bénard, F. Preclinical Melanoma Imaging with ^{68}Ga-Labeled α-Melanocyte-Stimulating Hormone Derivatives Using PET. *Theranostics* **2017**, *7*, 805–813.

7 Kuo, H.-T.; Lin, K.-S.; Zhang, Z.; Zhang, C.; Merkens, H.; Tan, R.; Roxin, A.; Uribe, C. F.; Bénard, F. What a Difference a Methylene Makes: Replacing Glu with Asp or Aad in the Lys-Urea-Glu Pharmacophore of PSMA-Targeting Radioligands to Reduce Kidney and Salivary Gland Uptake. *Theranostics* **2022**, *12*, 6179–6188.
